# The Clinical Features and Bacteriological Characterizations of Bone and Joint Tuberculosis in China

**DOI:** 10.1038/srep11084

**Published:** 2015-06-08

**Authors:** Su-Ting Chen, Li-Ping Zhao, Wei-Jie Dong, Yun-Ting Gu, Yun-Xu Li, Ling-Ling Dong, Yi-Feng Ma, Shi-Bing Qin, Hai-Rong Huang

**Affiliations:** 1 National Clinical Laboratory on Tuberculosis, Beijing Key Laboratory for Drug-resistant Tuberculosis Research; 2 Department of Orthopaedics, Beijing Tuberculosis and Thoracic Tumor Institute, Beijing Chest Hospital, Capital Medical University, Beijing China 101149

## Abstract

Bone and Joint tuberculosis (BJTB) constitutes about 10% of total extra-pulmonary TB cases. Since the BJTB is a paucibacillary condition, there has been no systematic study on the bacterial characterization, especially the epidemiological feature. Here we collected the mycobacterial clinical isolates, analyzed the clinical features and the bacteriological characteristics from 113 BJTB cases reported in China. The mean age of the cases was 40.33 years while most of the patients fell into the 20–29 year age group; local pain was the most common onset symptom of BJTB cases; mean time from symptom onset to BJTB diagnosis was 13.16 months. 31 isolates were defined as drug resistant, including 15 multidrug resistant (MDR) and 2 extensively drug resistant (XDR) isolates according to the drug susceptibility test outcomes; after spoligotyping, 87.6% (99/113) isolates were categorized as Beijing family. In contrast to the isolates from pulmonary tuberculosis patients, here the MIRU-VNTR assay did not find anything significant. A prolonged time span for BJTB diagnosis highlights the requirement of paying further attention to BJTB infection in China. This study provides essential insights into the demographic and microbial characteristics of BJTB cases in China.

China is ranked among the the 22 countries with high tuberculosis (TB) burden and also hosts the second largest population of tuberculosis patients, thus far the understanding of extra-pulmonary TB in China remains limited. Among all extra-pulmonary TB cases, about 10% of are BJTB, which is one of the most common infection sites 1,2,3,4. However, the clinical features and bacteriological characterization of bone and joint tuberculosis (BJTB) remain unclear as the current research mainly includes the case reports.

Beijing Chest Hospital has been the most influential institution on TB patients’ health care in China, it provides medical services for over 200,000 TB suspects per year whereas the department of orthopedics in the hospital treats more than 500 BJTB patients per year. A retrospective analysis was carried out on the demographic and clinical features of 113 culture confirmed BJTB patients. Additionally, the bacterial characterizations of the isolates were also performed since, to the best of our knowledge, for BJTB these features have not been reported so far. The present study was conducted to gain insights into the demographic and microbial characteristics of BJTB cases in China, with a view to extend the knowledgebase of BJTB for developing better TB control strategies.

## Methods

### Study setting and design

From January 2011 to December 2012, specimens were collected from the bone, tissue and pus during skeletal operations. Each of the specimens was subjected to solid Löwenstein-Jensen (LJ) and BACTEC MGIT 960 culturing and the culture confirmed BJTB cases were undertaken in our assay. Specimens were collected from a total of 206 patients, among them 113 cases acquired positive culture outcomes. For any patient with multiple isolates from the culture, only the first isolate was used for performing the bacterial characterization. The medical records of the enrolled patients were retrospectively reviewed and their demographic features and clinical information (including sex, age, locations affected, onset symptom, previous of TB, HIV status, diabetes and history of trauma, *et al.*) were collected. Informed consent was obtained from all subjects included in the study. The protocols and procedures for the protection of human subjects were approved by the Ethics Committee of Beijing Chest Hospital, furthermore, all the methods were carried out in accordance with the approved guidelines.

### Culture

Both LJ and MGIT 960 cultures were performed following our standard laboratory protocol. Briefly, the specimen was decontaminated and diluted by treating with an equal volume of 2% sodium hydroxide and 0.5% NALC (N-acetyl-l-cysteine-sodium hydroxide) for 15 min. After dilution, the tube was adjusted with 50 ml of 0.1 M phosphate buffer (pH 6.5) and centrifuged at 4000 × g for 20 min. Pellets were re-suspended in 20 ml of the phosphate buffer, re-centrifuged and the final pellet was re-suspended in 1 ml phosphate buffer to allow enough volume for solid LJ culture and liquid culture in the MGIT 960 system. Approximately 0.5 ml of deposit was inoculated into/onto each culture media.

### Species identification

16 s rRNA gene and 16 s–23 s rRNA internal transcribed spacer (ITS) regions were amplifiied by a Polymerase Chain Reaction (PCR) and subsequently sequenced for categorizing the isolates into species as mentioned elsewhere^5^,^6^.

### Drug susceptibility testing (DST)

The drug susceptibility test was performed for these isolates against four first-line anti-TB drugs [isonazid (INH), rifampin (RIF), ethambutol (EMB) and streptomycin (Sm)] and 7 second-line anti-TB drugs (kanamycin, amikacin, capreomycin, ofloxacin, levofloxacin, prothionamide, p-aminosalicylic acid). The protocol and the critical concentrations of drugs in the media were used as recommended by WHO/IUATLD^7^. Isolates resistant to both INH and RIF were defined as MDR-TB, while besides INH and RIF, isolates showing resistance to any member of the quinolone family and at least one of the second-line anti-TB injectable drugs were defined as XDR-TB. Pre-XDR is defined as MDR-TB plus resistance to either a fluoroquinolone drug or an aminoglycoside drug, but not both simultaneously.

### Spacer oligonucleotide typing (spoligotyping)

Genomic DNA was extracted by boiling the freshly cultured bacteria while PCR reaction and hybridization process were done as previously described^8^. A commercially available kit for spoligotyping (Isogen Bioscience BV, Maarssen, Netherlands) was used according to manufacturer’s instructions. Beijing genotype strains were defined with the pattern that hybridized to all of the last nine spacer oligonucleotides (spacers 35 to 43), and Beijing-like genotype strains were the ones that hybridized to only some of the last nine spacers^9^.

### MIRU-VNTR genotyping

The classical 24 VNTR loci were amplified by PCR as described previously^10^,^11^. Briefly, the PCR was performed using locus-specific primers and the number of repeats at each locus in the PCR product was interpreted by the Quantity One software (Bio-Rad) based on the electrophoretic mobility of the products compared with the standard molecular marker (50 bp DNA Ladder Marker, TaKaRa, China). Alleles were assigned numerical values according to the number of repeats present at a specific genomic locus. Isolates were classified as different genotypes according to MIRU-VNTR locus set while isolates having identical MIRU-VNTR profile type were defined as a cluster. MIRU-VNTR dendrograms were constructed by using unweighted pair group method with arithmetic averages (UPGMA).

### Statistical analysis

The database comprising of all the genotyping and DST results and epidemiological investigation information was created with PASW^®^statistics11 (Chicago, USA). The clustering rate was defined as (*n*_c_-*c*)/*n*, where *n*_c_ was the total number of the isolates clustered, *c* was the number of clusters, and *n* is the total of isolates used in the study.

## Results

**Figure 1:**
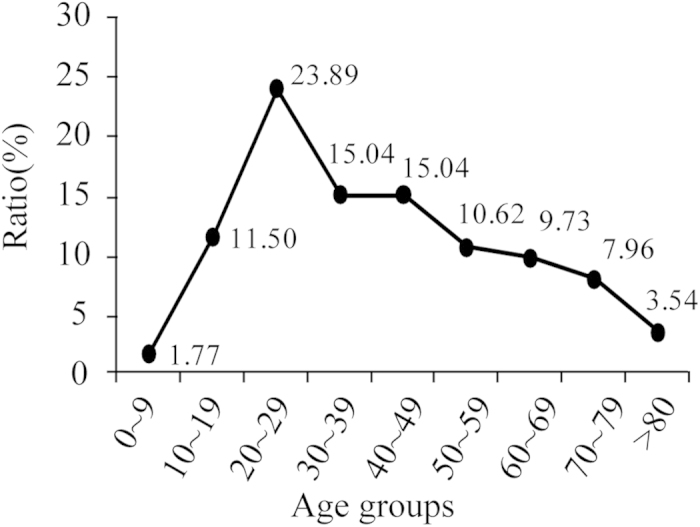


Among the 113 BJTB patients, 67 were male and 46 were female, male to female ratio was 1.457; the mean age was 40.33 years old (range 6–84, med 36). The largest number of patients fell into the 20–29 year age group (23.89%) followed by 30–39 year age group (15.04%) and 40–49 year age group (15.04%) (Fig. 1). The differences male and female patients’ age distributions, drug resistance and sites of infection are shown in Table 1. Overall, the age distributions and drug resistance profile among male and female patients demonstrated significant differences. A significant age difference between patients with different sex was found in this assay (mean age, 37.0 vs. 49.9 years; *P* = 0.001). Among patients aged <60 years, BJTB prevalence was significantly higher among males than females (OR, 6.03; 95% CI, 2.27–16.05; *P* < 0.001). Only 20 patients were the locals of Beijing area, while the other 93 lived outside of Beijing. Although the place of residence of the patients included 19 other provinces of China, their majority lived in northern China. Nine patients had immunosuppressive diseases that included 4 diabetes, 2 anaphylactoid purpura, 1 rheumatoid arthritis, 1 systemic lupus erythematosus, 1 pneumoconiosis cases. Nine patients had the history of osteoarticular trauma, and among them, 4 were affected at the current BJTB infection site.

The mean time from symptom onset to being diagnosed as BJTB was 13.16 months (range from 0.5 to 96 months, median delay was 7 months). Before the surgeries, histopathological evidences were demonstrated that only 5 out of the 113 patients were explicitly BJTB cases, while the other 108 patients were empirically diagnosed by means of imaging tests like CT (computed tomography) or nuclear magnetic resonance (NMR). 18 patients had a previous history of TB, including 9 BJTB, 2 pulmonary tuberculosis (PTB), 3 pleural tuberculosis and 4 other cases with different infection sites. 16 patients (15.04%) had concomitant TB, including 7 PTB, 3 lymphoid tuberculosis, 3 pleural tuberculosis and 3 Chest Wall Tuberculosis Abscess. The affected bone and joint locations of the recruited patients are listed in table 2. Spine was the most frequently affected location among these BJTB patients, covering 69.17% of the cases.

All the cases in this study were symptomatic. Local pain (83.18%, 94/113) was the most common onset symptom of the BJTB cases, followed by swelling of the affected part (11.5%, 13/113) and impairment of function (3.5%, 4/113). Systemic symptom such as fever, night sweat and weight loss was the onset symptom of 1.76% (2/113) cases. Commonly, only a single affected site observed but 20 (17.7%) patients demonstrated multiple affected locations, whereas 16 out of those 20 patients had more than one lesion on spine. No statistically significant difference was observed between the male and female populations with respect to the affected locations.

All of the 113 clinical isolates were identified as *Mycobacterium tuberculosis* complex (MTC) by 16 s rRNA gene and ITS sequencing. Among these 113 isolates, 82 (72.6%) had pan-sensitive DST features, 31 (27.4%) were resistant to at least one anti-tuberculosis drug, including 5 single drug resistant (2 for INH, 2 for Sm, 1 for protionamide), 11 poly drug resistant and 15 (13.3%) were multidrug resistant (MDR) isolates. 2 MDR isolates could be further defined as extensively drug resistant (XDR) isolates according to the DST outcomes. We found that 11 isolates were resistant to INH but still sensitive to RIF, while none of the isolates to be resistant to RIF while still sensitive to INH. In addition, the percentage of INH, or RIF, or Sm-resistant and MDR-TB isolates was significantly higher among male patients than among female patients (*P* < 0.05) (Table 1).

No *M. bovis* was identified by spoligotyping. Among the recruited isolates, a total of 99 (87.6%) isolates were identified as the Beijing genotype, while 14 (12.4%) were from the non-Beijing families. Isolates classified into non-Beijing families included 5 isolates from the T1 family (4.4%), 1 from the MANU3 family (0.9%), 1 from the T2 family (0.9%), 1 from the T3 family (0.9%), and 6 of undefined spoligotype genotypes (5.3%) (Table 3). For the 113 *M. tuberculosis* isolates genotyped, a total of 18 spoligotypes were identified. Cluster analysis revealed that SIT 1 was the largest lineage (80.5%), belonging to the classical Beijing genotype, followed by SIT 190 (n = 3 [2.7%]) (Table 3). Moreover, 2 single-drug resistant isolates (both resistant to Sm) and 1 poly drug resistant isolate belonged to the non-Beijing families, while all other drug resistant strains (including 4 mono-drug resistant isolate, 11 poly resistant isolates, 15 MDR isolates and 2 XDR isolates) had Beijing genotypes. The percentage of drug resistant isolates seemed higher among Beijing family than non-Beijing family, but the difference was not statistically significant (Data not shown).

In this study, only 105 isolates were successfully analyzed using the 24 loci MIRU-VNTR system for technical reasons, and each isolate exhibited unique MIRU-VNTR pattern, therefore the clustering rate with the 24 loci was zero. Among the 24 VNTR loci, the allelic diversity for MIRU31, QUB-11b, Mtub21, MIRU26 and Mtub04 was higher than 0.6, thus classified as the highly discriminating loci; while MIRU16, MIRU20, MIRU23, MIRU24 and MIRU02 had negligible diversity (HGDI < 0.1). The other loci showed intermediate discrimination power between these two groups (Table 4).

## Discussion

BJTB is the third most common infection of extra-pulmonary tuberculosis diseases after pleural and lymphatic TB^1^. However, as BJTB is a paucibacillary condition, no systematic study on the bacterial characterization, especially epidemiological features, has been completed.

Meanwhile, total 592 BJTB cases underwent treatment in Beijing Chest Hospital from January 2011 to December 2012, when this project was going on. The demographic and clinical features of the overall patients were analyzed. Among the 592 BJTB patients, 356 were male, 236 were female, and the male to female ratio was 1.508; the mean age was 44.35 years old (range 2–88, median age was 46). Of the 592 skeleton patients, 113 cases were microbiologically-confirmed. In comparison to the overall number of BJTB, the microbiologically-confirmed BJTB cases were 4 years younger, with a median age of 36 years (Supplementary Figure 1). But other than that, there was no significant difference between these two groups (Supplementary Table 1).

The onset of BJTB is usually an insidious process that often takes months and occasionally years from the first symptom to diagnosis. Among our recruited patients, the mean time from the first symptom onset to being diagnosed as BJTB was 13.16 months (range from 0.5 to 96 months, median delay was 7 months). A prolonged time span in our study, in contrast to others carried out in developed countries^1^,^12^,^13^,^14^, reflects the shortage of effective diagnosis measures and social-economic differences. Firstly, due to limited disease specific symptoms and the paucibacillary nature, BJTB could easily be diagnosed as some other skeleton disease. Secondly, poor people, who may be more vulnerable subjects for tuberculosis, often refuse to visit doctor until the appearance of functional damage. Although mycobacterial culturing remains the gold standard for TB diagnosis, the low recovery rate limits its role in BJTB diagnosis. Histopathological diagnosis is often problematic since the infection sites remain poorly accessible and patients tend to be less compliant towards invasive procedures^12^. Currently, CT and MRI are the mainstay for BJTB diagnosis^13^,^15^,^16^, but these are limited due to higher cost, inaccessibility to a lot of patients, and requirement of experienced image analysts. A majority (94.6%) of our recruited patients were empirically diagnosed as BJTB according to imaging tests, while the histopathological and microbiological evidences obtained after biopsies or surgeries confirmed the diagnosis.

Although a previous study reported that female patients were more than twice as likely as male patients to have extrapulmonary TB^17^, we failed to verify association between female gender and the BJTB disease. However, the male to female ratio in recruited patients was 1.457, which was much lower than that of Chinese PTB patients^18^. The gender inequalities might increase the male BJTB patient’s proportion in our study, as male patients may have more chance to access the expensive imageological examinations and operations. The most frequent age of the onset of BJTB was during the third decade (23.89%), followed by fourth (15.04%) and fifth decades (15.04%). BJTB more frequently affects older children and young adults in high TB burden countries, while in developed countries the disease is often seen in older persons^19^,^20^,^21^. In this study, we found that BJTB more frequently affected older children, young male adults, and old female adults (>59 years). For the number of females suffering was greater than that of male in elderly patients with BJTB, one plausible explanation is that women have longer life spans than men.

Among patients in our study, local pain was generally the first presenting symptom, accounting in 83.18% of the patients; followed by swelling of the affected part (11.5%) and impairment of function (3.5%). Only less than one third (34 out of 113) of the cases of BJTB had active or healed TB. Single site involvement was generally seen, but association of multiple locations also happened in 19 cases (16.8%). Vertebral TB was reported to be the most common type of BJTB, accounting for about 50% of cases in most series^22^,^23^,^24^. Among our patients, vertebral involvement accounted for about 70% of all cases. We attributed this high percentage to study population and methodological differences. For example, the difference of the inclusion criteria may lead to the difference. Among the vertebral TB patients, lumber vertebra was most affected location (48.91% of the group, 39.82% of the total cases), followed by thoracic vertebra (38.04% of the group, 22.12% of the total cases). TB can also cause disease of the peripheral joints, but these were less common manifestation. Hip joint was the most frequently affected location (36.84% of the group, 12.39% of the total cases), followed by knee (21.05% of the group, 7.08% of the total cases) (see Table 2).

All the isolates enrolled in this study belonged to *Mycobacterium tuberculosis* complex, and no *M. bovis* was identified by spoligotyping. The single drug resistance rate (29.1%) and MDR rate (13.27%) in the BJTB patients here was closer to that of newly treated PTB patients (33.9%, and 5.7%, respectively), but lower than that of retreated PTB patients (53.0% and 25.5%, respectively) in China. Although lengthy delay in diagnosis was commonly seen, no anti-TB treatment was launched before explicit diagnosis reduced the risk of drug resistance. Among the 15 MDR cases, 5 had concomitant TB, 5 had history of TB, while the other 5 had endured a long- time anti-TB treatment before isolate collection. This data reflected that acquired drug resistance might be the main reason for the prevalence of drug resistance among BJTB. We found 11 isolates were resistant to INH but still sensitive to RIF, while none of the isolates resistant to RIF while still sensitive to INH. Our results support the practice to use rifampicin resistance as a marker for MDR isolate screening, and also justify the usefulness of some rapid molecular tests like Xpert MTB/RIF in BJTB diagnosis. Although the isolates obtained after operation are less useful for the early diagnosis of BJTB, the DST outcome is still of great importance for chemotherapy regimen adjustment after operation.

According to other previous reports on pulmonary tuberculosis (PTB), the Beijing genotype accounts for 76.5% of the MTB isolates currently epidemic in northern China and 53.2% of those in southern China, with the proportions in provinces countrywide ranging from 52.53% to 92.59%^25^. Our results demonstrate that the Beijing genotype is highly prevalent among BJTB patients from Beijing and surrounding areas. Since the proportion was higher than most reports from northern China on PTB cases, thus, the Beijing genotype isolates may have some special biological features that facilitate the development of BJTB. Considering the correlation between Beijing genotype and drug resistance, controversial opinions were drawn by different authors for PTB patients^25^. In this study, we observed that no significantly higher proportion of drug resistance among Beijing genotype isolates than among non-Beijing genotypes (*P* > 0.05). However, since non-Beijing stains only account for a minority of all the isolates, data could be slightly biased. Other studies have analyzed the association between certain phylogenetic lineages and extrapulmonary tuberculosis but have shown conflicting results. Those assays were also limited by relatively small sampling size and phylogenetic diversity^26^,^27^,^28^,^29^,^30^.

We did not find any cluster among the recruited isolates by the 24 classical loci MIRU-VNTR analysis. We attributed this outcome to diverse residence area of the enrolled patients, and to the sporadic character of the disease. Considering the discriminative capability of each locus, the outcomes of this assay obtained from BJTB isolates were comparable with that from PTB isolates^31^,^32^,^33^. In this assay, 8% patients had immunosuppressive diseases; another 8% patient had histories of osteoarticular trauma, which indicated those situations might be the risk factors for BJTB. In consistence with other research, our study indicated that the host related-factors might be the main reason for BJTB development and not the pathogenic factor^15^.

Limitations of our study are worth noticing. We only enrolled culture confirmed BJTB cases, thus, our results might have a bias on patients with more severe disease. Although our patients come from 19 different provinces of China, the majority of patients lived around Beijing area. So our data and conclusion might only be representative of the area closer to Beijing, or northern China. Our work was limited by relatively small sample size, whereas obtaining representative data for China now is impossible because our nationwide TB registration system only focuses on PTB patients.

This is the first systemic report specific to the bacteriological characters of BJTB patients. In this assay, we analyzed the clinical features of BJTB patients in Beijing, China and studied the drug resistant patterns and genotypes of the isolates of those patients. A more lengthy delay of BJTB diagnosis showed that more attention to BJTB infection is needed in China. Beijing family strains of MTB were predominant in BJTB cases, whereas there was no correlation between the drug-resistance and Beijing family strains of BJTB. Our work is helpful to gain a better understanding of the epidemiology of BJTB and to improve the appropriate management of the disease.

## Additional Information

**How to cite this article**: Chen, S.-T. *et al.* The Clinical Features and Bacteriological Characterizations of Bone and Joint Tuberculosis in China. *Sci. Rep.* **5**, 11084; doi: 10.1038/srep11084 (2015).

## Supplementary Material

Supplementary Information

## Figures and Tables

**Table 1:** **Difference of characteristics between male and female patients with BJTB.**

**Characteristic**	**Male**	**Female**	**Odds ratio**	***P* value**
	**(n = 67)**	**(n = 46)**	**(95%CI)**	
	**No. (%)**	**No. (%)**		
Age group				
<20	8 (11.9)	2 (4.3)	10.86 (1.84–64.08)	0.007
20–39	31 (46.3)	15 (32.6)	5.61 (1.94–16.25)	0.001
40–59	21 (31.3)	10 (21.7)	5.70(1.81–17.97)	0.002
0–59	60 (89.6)	27 (58.7)	6.03 (2.27–16.05)	0.000
>59	7 (10.4)	19 (41.3)	1.0 (reference)	—
Resistance to				
INH	22 (32.8)	7 (15.2)	2.72 (1.05–7.06)	0.035
RMP	14 (20.9)	2 (4.3)	5.81 (1.25–26.96)	0.013
SM	23 (34.3)	4 (8.7)	5.49 (1.75–12.21)	0.002
EMB	0 (0)	0 (0)	0	0
MDR	13 (19.4)	2 (4.3)	5.30 (1.13–24.73)	0.02
Pre-XDR	2 (3.0)	0 (0)	0	0.513
Affected location				
Spine	43 (64.2)	33 (71.7)	0.71 (0.31–1.59)	0.400
Others	24 (35.8)	13 (28.3)	1.0 (reference)	—
Immune diseases				
HIV	0 (0)	0 (0)	—	—
Diabetes	0 (0)	4 (8.7)	—	—
Anaphylactoid purpura	2 (3.0)	0 (0)	—	—
Rheumatoid arthritis	1 (1.5)	0 (0)	—	—
Systemic lupus erythematosus	0	1 (2.2)	—	—
Pneumoconiosis	1 (1.5)	0 (0)	—	—
Osteoarticular trauma	4 (6.0)	5 (10.9)	—	—

**Table 2:** **Anatomic site of musculoskeletal TB among the 113 cases** *.

**Group**	**Site**	**No.**	**% of group**	**% of total**
I. Spine (n = 92 ) 69.17% of total	Cervical	2	2.17	1.78
	Thoracic	35	38.04	22.12
	Lumbar	45	48.91	39.82
	Sacrum	10	10.87	8.85
II. Peripheral Joints (n = 38 ) 28.57% of total	Hip jont	14	36.84	12.39
	sacroiliac joint	2	5.26	1.78
	symphysis pubis	2	5.26	1.78
	Knee	8	21.05	7.08
	Ankle	3	7.89	2.65
	Foot	3	7.89	2.65
	Shoulder	2	5.26	1.78
	Elbow	3	7.89	2.65
	Wrist	1	2.63	0.88
III. Other (n = 3)	sternoclavicular	3	100	2.65

^*^number in the tables indicates affected organs, not the cases.

**Table 3:** **The Spoligotyping distribution of 113 clinical isolates of BJTB patients according to SITVITWEB database.**

**Spoligotyping**	**SIT**	SpolDB4 ID*	**Isolate No.**	**Prevalence (%)**
000000000003771	1	Beijing	91	80.5
000000000003731	190	Beijing	3	2.7
000000000000071	orphan	Beijing	2	1.8
000000000003471	1364	Beijing	1	0.9
000000000003761	1674	Beijing	1	0.9
000000000002771	621	Beijing	1	0.9
777377777770771	orphan	MANU3	1	0.9
777777777760771	53	T1	2	1.8
777777777760000	1793	T1	1	0.9
777777677560771	453	T1	1	0.9
777777707760371	2069	T1	1	0.9
777777777760731	52	T2	1	0.9
777737777760711	1426	T3	1	0.9
777777770000000	46	NA	1	0.9
000000000003611	New	NA	1	0.9
000000000003741	New	NA	2	1.8
777740000003771	NEW	NA	1	0.9
000000000000761	New	NA	1	0.9

^*^NA represents the spoligotyping type which is not found in SITVITWEB database.

**Table 4:** **General information of VNTR 24-locus set and comparison of resolution in 105 clinical isolates of BJTB patients** #.

	**Times of repeat**												
**Loci**	**0**	**1**	**2**	**3**	**4**	**5**	**6**	**7**	**8**	**9**	**10**	**11**	HGDI*
ETRC	0	0	1	4	99	0	1	0	0	0	0	0	0.110
MIRU16	0	0	4	100	1	0	0	0	0	0	0	0	0.092
Mtub39	0	2	2	90	7	4	0	0	0	0	0	0	0.261
Mtub30	1	0	8	1	94	1	1	0	0	0	0	0	0.194
MIRU40	0	2	9	88	6	0	0	0	0	0	0	0	0.289
MIRU31	0	0	5	7	33	55	5	0	0	0	0	0	0.623
QUB-11b	0	0	16	9	32	43	5	0	0	0	0	0	0.713
MIRU20	0	4	101	0	0	0	0	0	0	0	0	0	0.074
Mtub21	0	10	0	14	40	35	6	0	0	0	0	0	0.721
QUB26	0	0	0	1	3	3	5	20	64	7	2	0	0.589
Mtub34	0	0	1	88	16	0	0	0	0	0	0	0	0.277
ETR-B	0	3	59	43	0	0	0	0	0	0	0	0	0.521
MIRU23	0	0	0	0	0	104	1	0	0	0	0	0	0.019
MIRU39	0	0	10	93	2	0	0	0	0	0	0	0	0.208
MIRU27	0	0	6	95	4	0	0	0	0	0	0	0	0.178
MIRU02	0	0	105	0	0	0	0	0	0	0	0	0	0
MIRU24	1	104	0	0	0	0	0	0	0	0	0	0	0.019
Mtub29	1	0	1	6	97	0	0	0	0	0	0	0	0.145
MIRU26	0	0	0	1	22	13	11	51	4	1	1	1	0.699
MIRU10	0	2	17	85	1	0	0	0	0	0	0	0	0.321
Mtub04	0	4	19	13	60	9	0	0	0	0	0	0	0.623
MIRU04	0	2	8	84	6	4	0	1	0	0	0	0	0.352
ETR-A	0	0	2	18	84	0	1	0	0	0	0	0	0.333
QUB-4156c	0	1	6	83	6	9	0	0	0	0	0	0	0.365

^*^HGDI: Hunter-Gaston Discrimination Index; HGID = 1 − [Σn_*j*_(n_*j*_ − 1)]/N(N − 1); N is the total number of strains in the sample population, s is the total number of types described, and n_*j*_ is the number of strains belonging to the *j*th type.

^#^Only 105 isolates were successfully analyzed using the 24 loci MIRU-VNTR system.
