# Supplementary material for: The Clinical Features and Bacteriological Characterizations of Bone and Joint Tuberculosis in China
Source: Sci Rep. 2015 Jun 8;5:11084. doi: 10.1038/srep11084 (PMC4459174; doi:10.1038/srep11084)
Supplement: Supplementary Information [file srep11084-s1.pdf]

The Clinical Features and Bacteriological Characterizations of Bone and Joint Tuberculosis in China

Su-Ting Chen,[1] Li-Ping Zhao,[1] Wei-Jie Dong,[2] Yun-Ting Gu,[1] Yun-Xu Li,[1] Ling-Ling Dong,[1] Yi-Feng Ma,[1] Shi-Bing Qin,[2] Hai-Rong Huang[1*]

1. National Clinical Laboratory on Tuberculosis, Beijing Key laboratory on Drug-resistant Tuberculosis; 2. Department of Orthopaedics, Beijing Tuberculosis and Thoracic Tumor Institute, Beijing Chest Hospital, Capital Medical University, Beijing China 101149

Running title: Characterizations of Bone and Joint Tuberculosis in China

---

[*]Correspondence should be addressed to Hairong Huang (hairong.huangcn@gmail.com). Mailing address: 97 Beimachang, Tongzhou District, Beijing, 101149; Tel: 8610-89509359; Fax: 8610-89509359.

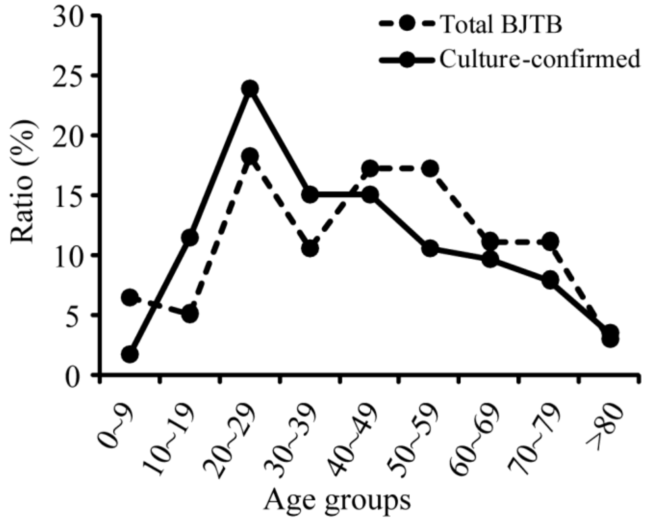

Figure S1. Difference of age distribution between all the BJTB patients and culture-confirmed BJTB patients.

Table S1. Difference of characteristics between male and female patients with culture-confirmed and not culture-confirmed BJTB

**Characteristic**	**Male (n=356) n (%)**	**Female (n=236) n (%)**	**Odds ratio (95%CI)**	***P* value**
Age group				
<20	64	4	28.44 (9.82–82.41)	0.000
20–39	118	52	4.03 (2.53–6.43)	0.000
40–59	120	84	2.54 (1.64–3.92)	0.000
0–59	302	140	3.84 (2.60–5.66)	0.000
>59	54	96	1.0 (reference)	0.000
Affected location				
Spine	222	176	0.57 (0.39–0.81)	0.002
Others	134	60	1.0 (reference)	—
Immunosuppressive diseases				
HIV	0	0	—	—
Diabetes	22	32	—	—
Anaphylactoid purpura	2	2	—	—
Rheumatoid arthritis	2	0	—	—
Systemic lupus erythematosus	0	2	—	—
Pneumoconiosis	2	0	—	—
Osteoarticular trauma	8	10	—	—
